# Supplementary material for: Signal-Retaining Autophagy Indicator as a Quantitative Imaging Method for ER-Phagy
Source: Cells. 2023 Apr 11;12(8):1134. doi: 10.3390/cells12081134 (PMC10136497; doi:10.3390/cells12081134)
Supplement: Supplementary file 1 [file cells-12-01134-s001.zip › Supplementary figures.pdf]

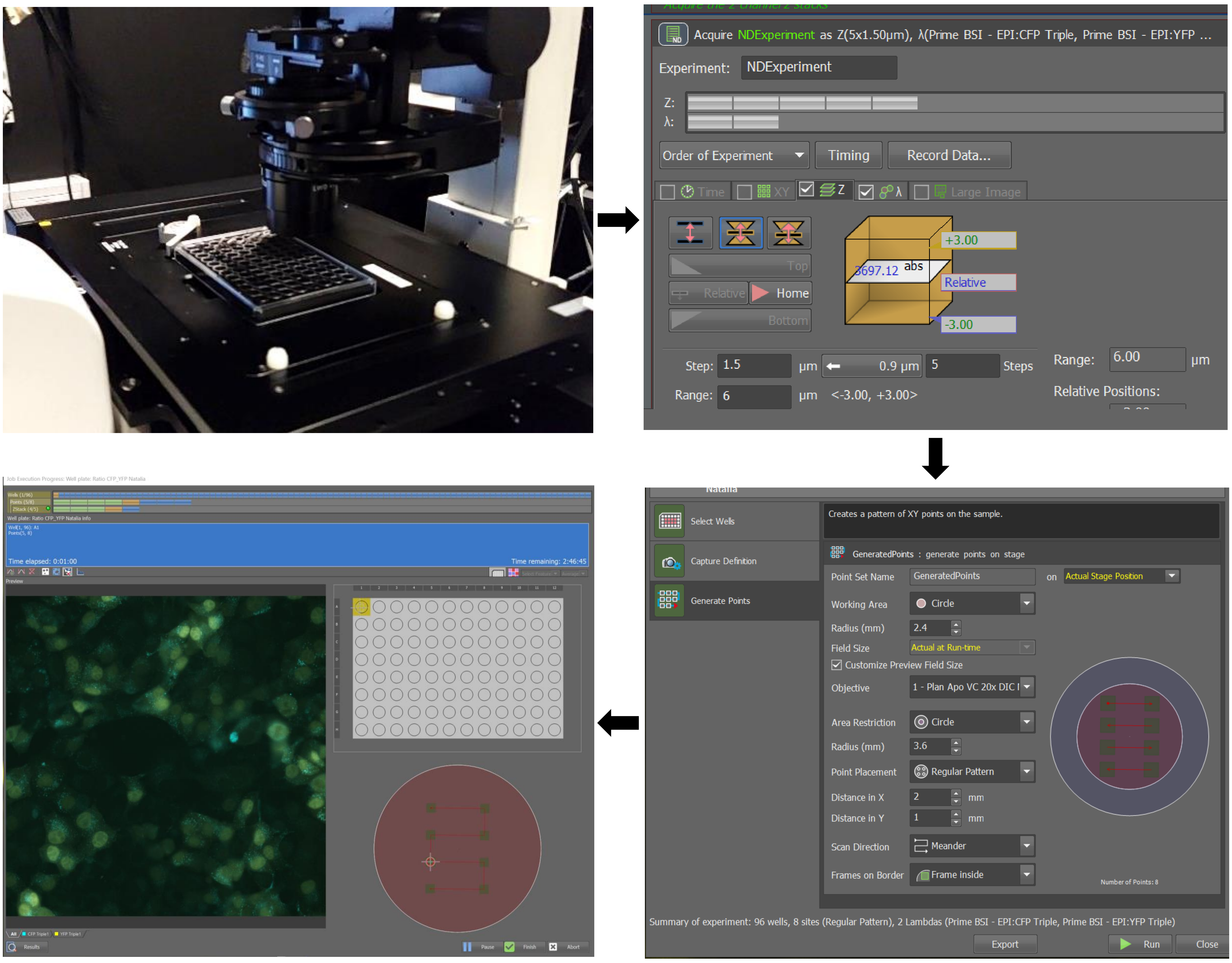

Supplementary Figure S1. Workflow for conditional epifluorescence imaging of SRAI reporters. Diagram representing workflow of automated imaging in a 96-well plate format. 96 well plates were imaged in an inverted wide-field microscope and Nikon Nis-Elements JOBS was used to set up and control image acquisition of TOLLES (CFP filter) and YPet (YFP filter) channels.

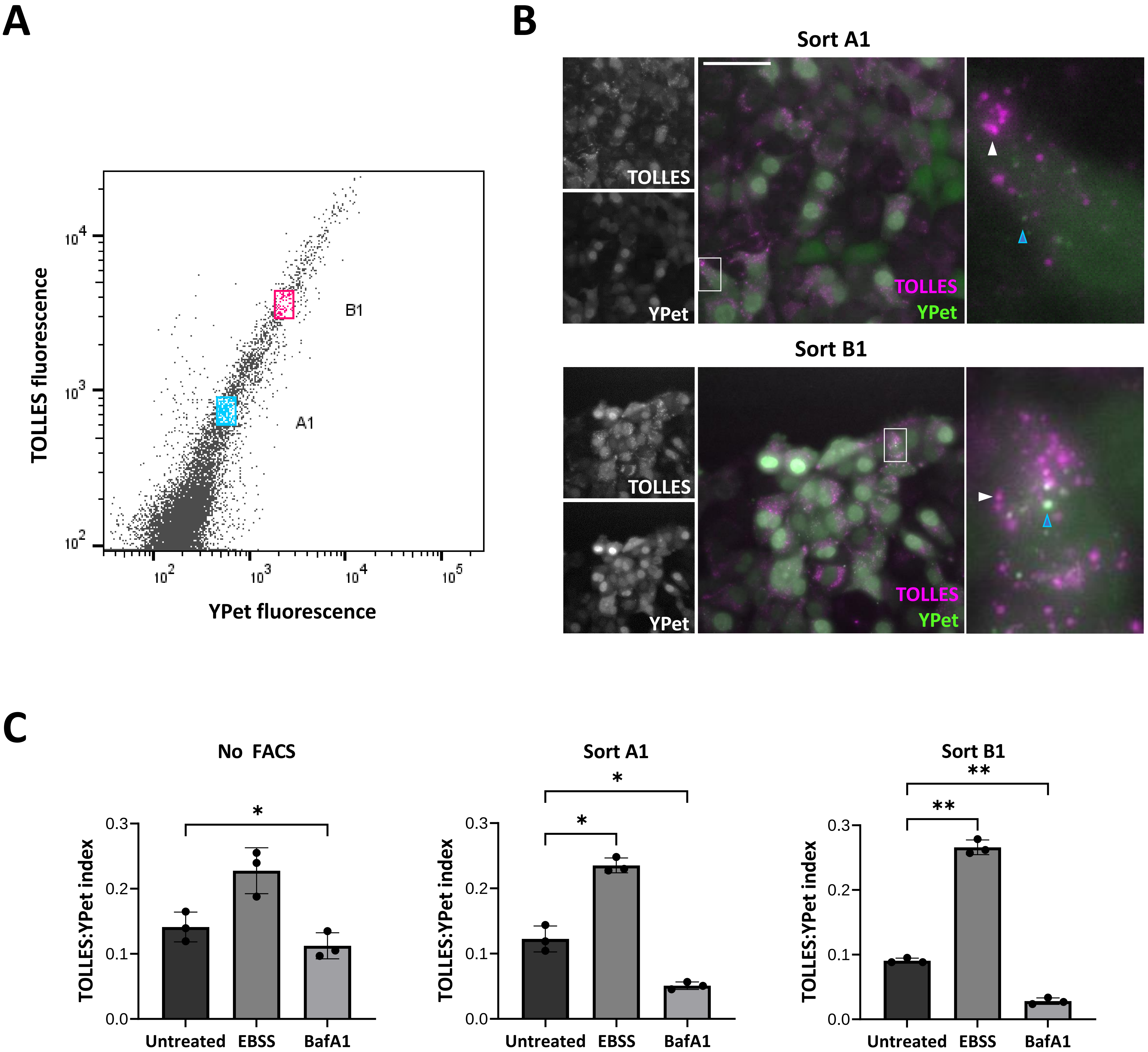

Supplementary Figure S2. Sorting of SRAI-expressing cells improves TOLLES:YPet index quantification. (**A**) FACS sorting of PDAC cells stably expressing SRAI-LC3B using narrow gates to select medium/high-TOLLES-YPet expression cell populations (A1 and B1). (**B**) Representative immunofluorescence images of TOLLES (magenta) and YPet (green) and imaged by automated wide-field microscopy from PDAC cells stably expressing SRAI-LC3B after FACS sorting from A; white arrows show examples of TOLLES-positive and YPet-negative puncta and blue arrows indicate TOLLES and YPet-positive puncta. Bar = 50 µm. (**C**) Quantification of the TOLLES:YPet index from non-sorted or sorted PDAC cells stably expressing SRAI-LC3B in full nutrient (untreated), after 4 h nutrient deprivation or after 50 nM BafA1 treatment (n = 3, 6 fields per replicate were analysed with 60-100 cells per field, ± S.D., * = $p < 0.05$, ** = $p < 0.01$, 1-way ANOVA followed by Holm-Šídák comparisons versus untreated control).

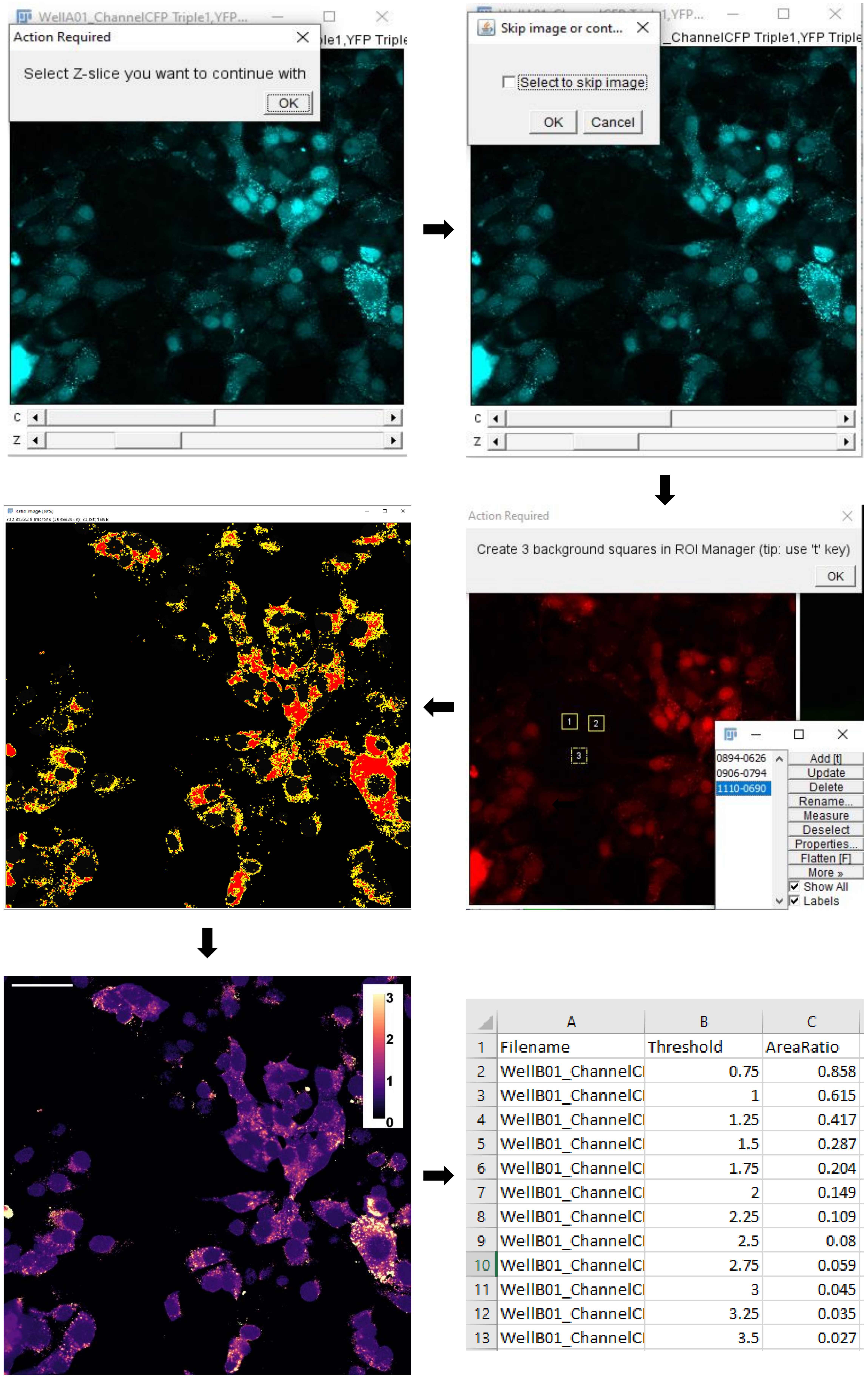

Supplementary Figure S3. TOLLES:YPet index workflow. Semi-automated quantification of autophagy flux using the ImageJ TOLLES:YPet index macro. Captured images using automated microscopy will be used as user input is needed to select the best representative z-slice, decide if to discard the image and select background ROIs to subtract the value from each channel. Then, the macro will analyse the ratio of the two channels using different thresholds that can be modified in the script and will generate the ratio images and calculate the TOLLES:YPet index. Bar = 50 µm.
